# S41. Novel CEA-targeted IL2 variant immunocytokine for immunotherapy of cancer

**DOI:** 10.1186/2051-1426-2-S2-I8

**Published:** 2014-03-12

**Authors:** C Klein

**Affiliations:** 1 Roche Glycart AG, Schlieren, Switzerland

## Background

Here we describe a novel class of monomeric tumor-targeted immunocytokines that comprise a single IL-2 variant (IL2v) with abolished CD25 binding that is fused to the C-terminus of a tumor specific antibody with a heterodimeric Fc devoid of FcγR and C1q binding. For tumor targeting, human/humanized high affinity antibodies against CEA (GA504) or FAP (GA501) were selected.

## Materials & methods

CEA- and FAP-IL2v activity was tested on effector cells by assessing the activation of P-STAT5, cell proliferation, sensitivity to Fas-induced apoptosis, expression of activation markers and cytokine release upon treatment. Safety, pharmacokinetics, pharmacodynamics and anti-tumor efficacy were analyzed in fully immunocompetent (CEA transgenic) C57Bl/6 mice as single agent and in combination with ADCC competent antibodies in SCID/hCD16 tg mice as well as Balb/neuT genetically engineered mice. Tumor targeting was investigated in the orthotopic syngeneic Renca RCC model in Balb/c mice.

## Results

FAP- and CEA-IL2v completely lack binding to CD25, but retain IL-Rβγ binding, and show pM binding affinity to respective antigens, FAP or CEA. As a consequence of abolished binding to CD25 they do not preferentially activate Tregs, but IL-2Rβγ mediated activity is retained and FAP- and CEA-IL2v activate NK, CD4^+^ and CD8^+^ T cells as shown by induction of activation markers, cell proliferation and cytokine release. Furthermore, CEA-IL2v and FAP-IL2v enhance the cytotoxic activity of NK cells when combined with ADCC-competent antibodies. Mechanism of action studies in fully immunocompetent mice showed that the molecules strongly expand and activate NK, CD8^+^ T cells and gd T cells (up to 100-fold) and skew the CD4:CD8 ratio strongly towards CD8^+^ T cells in the peripheral blood, lymphoid tissues, and in the tumor. In C57Bl/6 mice. MicroSPECT/CT imaging with radioactively labeled FAP-IL2v reveal good FAP-mediated tumor targeting in the orthotopic syngeneic Renca model with low normal tissue uptake and low accumulation in lymphoid tissues, contrary to analogous IL-2 based immunocytokine that shows preferential targeting to lymphoid tissue. Studies in tumor-bearing mice show dose-dependent anti-tumor efficacy of CEA-IL2v in syngeneic MC38-CEA and PancO2-CEA models. Combination of a tumor-stroma targeted TNCA-IL2v with an ADCC-competent ratHER2 antibody in the Balb/neuT spontaneous breast cancer model results in enhanced antitumoral efficacy.

## Conclusion

Compared to classical IL-2-based immunocytokines, CEA- and FAP-IL2v demonstrate superior safety, PK and tumor targeting due to abolished CD25 binding, monovalency and high-affinity to tumor antigens while failing to preferentially induce Tregs. CEA- and FAP-IL2v retain the capacity to activate and expand NK and CD8+ effector T cells both in the periphery and tumor microenvironment supporting their further nonclinical and clinical investigation for immunotherapy of cancer. Clinical trials with CEA-IL2v are foreseen in 2014.

